# The Diverse Applications of Pancreatic Ductal Adenocarcinoma Organoids

**DOI:** 10.3390/cancers13194979

**Published:** 2021-10-04

**Authors:** Ronnie Ren Jie Low, Wei Wen Lim, Paul M. Nguyen, Belinda Lee, Michael Christie, Antony W. Burgess, Peter Gibbs, Sean M. Grimmond, Frédéric Hollande, Tracy L. Putoczki

**Affiliations:** 1 The Walter and Eliza Hall Institute of Medical Research, Melbourne, VIC 3052, Australia; low.r@wehi.edu.au (R.R.J.L.); lee.b@wehi.edu.au (B.L.); christie.m@wehi.edu.au (M.C.); tburgess@wehi.edu.au (A.W.B.); gibbs.p@wehi.edu.au (P.G.); 2 Department of Medical Biology, The University of Melbourne, Melbourne, VIC 3052, Australia; 3 Victorian Comprehensive Cancer Centre, University of Melbourne Centre for Cancer Research, Melbourne, VIC 3000, Australia; wei.lim@unimelb.edu.au (W.W.L.); paul.nguyen1@unimelb.edu.au (P.M.N.); sean.grimmond@unimelb.edu.au (S.M.G.); frederic.hollande@unimelb.edu.au (F.H.); 4 Department of Medical Oncology, Western Health, Melbourne, VIC 3052, Australia; 5 Department of Clinical Pathology, University of Melbourne, Melbourne, VIC 3000, Australia

**Keywords:** cancer, coculture, drug screen, fibroblast, immune, microenvironment, organoid, patient derived, PDAC, therapeutic

## Abstract

**Simple Summary:**

Patients diagnosed with pancreatic cancer have very few treatment options. In order to identify new treatment opportunities, and develop new drugs for clinical use, appropriate model systems that take into account the complexities of a tumor are required. In this review, we summarize the current and emerging opportunities to accurately model pancreatic cancer using organoid technologies. We highlight the need for continued development of these complex model systems in order to inform personalized treatment.

**Abstract:**

Pancreatic ductal adenocarcinoma (PDAC) is one of the most lethal solid malignancies. While immortalized cancer cell lines and genetically engineered murine models have increased our understanding of PDAC tumorigenesis, they do not recapitulate inter- and intra-patient heterogeneity. PDAC patient derived organoid (PDO) biobanks have overcome this hurdle, and provide an opportunity for the high throughput screening of potential new therapies. This review provides a summary of the PDAC PDO biobanks established to date, and discusses how they have advanced our understanding of PDAC biology. Looking forward, the development of coculturing techniques for specific immune or stromal cell populations will enable a better understanding of the crosstalk that occurs within the tumor microenvironment, and the impact of this crosstalk on treatment response.

## 1. Introduction

Pancreatic cancer is one of the most aggressive and deadliest cancer types, with a mortality-to-incidence ratio of 94%, an abysmal 5-year survival rate of less than 10%, and very little improvement in patient outcomes over the last decade [1,2]. Pancreatic cancer is the seventh leading cause of cancer-associated mortality, and it is alarming that pancreatic cancer incidence is increasing and predicted to be the second leading cause of cancer associated deaths in the next decade [1,3].

### 1.1. Pancreatic Ductal Adenocarcinoma, the Most Common Pancreatic Cancer

Pancreatic ductal adenocarcinoma (PDAC) accounts for 85% of pancreatic cancers [4]. The most frequent genetic change associated with the progression of PDAC include activating mutations in *KRAS*, which are present in 35% of low grade pancreatic intraepithelial neoplasias (PanIN), 75% of high grade PanIN, and in more than 90% of PDACs [5,6]. High grade PanIN can also have epigenetic and somatic alterations in *TP53* (92%), *CDKN2A* (31%), and *SMAD4* (12%) [7], while PDAC is also associated with alterations in *p16/CDKN2A* (>95%), *BRCA2* (10%), *TP53* (75%), and the TGF-β pathway components *SMAD4* (55%), *TGF-βRI* (<5%), *TGF-βRII* (<5%) [5]. Approximately 10% of PDACs arise from an alternative, genome-wide catastrophic event, termed ‘punctuated evolution’ or ‘chromothripsis’ [8], where thousands of structural alterations occur on one or a few chromosomes in a single cell cycle that results in structural damage and rearrangements in multiple driver genes [8]. 

There are four PDAC genomic subtypes, based on the structural variants present within the genome of the primary tumor. These include a ‘stable’ subtype, which has less than 50 structural variants and widespread aneuploidy; a ‘locally rearranged’ subtype, which is defined by more than 50 events localized to one or two chromosomes, often including amplified *KRAS*, *SOX9,* and *GATA6* [9]; a ‘scattered’ subtype that is characterized by 50 to 200 structural variants; and an ‘unstable’ subtype, which includes tumors with more than 200 structural variants [9]. 

**Figure 1:**
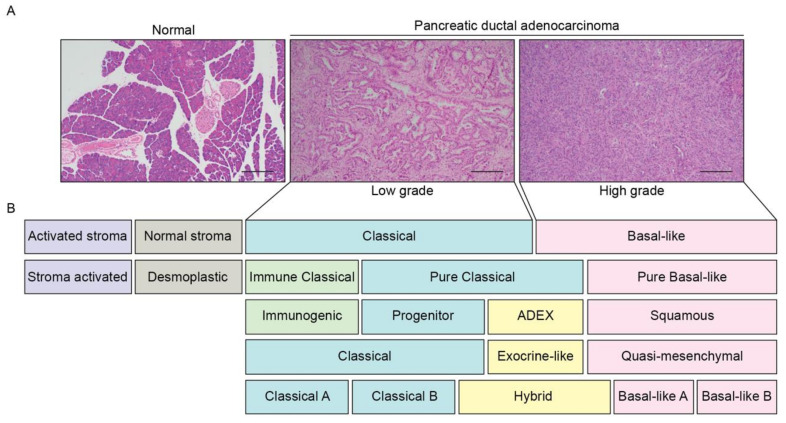
PDAC patients can be subclassified based on histopathological and molecular features. (**A**) Representative H&E sections of a normal human pancreas and a primary PDAC tumor (low grade and high grade). Scale: 200 μm. (**B**) Schematic overview of the PDAC molecular subtypes described to date [14,15,16,19,20]. Subtypes with similar expression signatures are grouped by color. Low grade PDAC tumors are enriched for a ‘classical’ signature, whereas high grade tumors are enriched for a ‘basal-like’ signature.

Histopathological assessment of PDAC involves a two-tiered grading system along with staging [10]. Low grade PDACs have well-formed glandular structures embedded within desmoplastic stroma with few mitoses and relatively mild nuclear pleomorphism [11], while in high grade tumors, single tumor cells infiltrate the stroma, or form poorly ordered sheets of cells with frequent mitoses and nuclear atypia (Figure 1A) [11]. In general, low grade tumors are slow growing with a favorable prognosis [12], while high grade tumors are associated with poor outcomes [12]. 

PDAC can also arise from cystic lesions, which include intraductal papillary mucinous neoplasms (IPMN), mucinous cystic neoplasms, intraductal oncocytic papillary neoplasms, and intraductal tubulopapillary neoplasms [13]. These less common premalignant tumors have distinct histological features, and molecular profiles that differ from PanIN associated PDACs, for example, *GNAS* and *RNF43* mutations are found in IPMN, *RNF43* mutations are present in mucinous cystic neoplasms, and *PIK3CA* and *PTEN* mutations are present in intraductal tubulopapillary neoplasm [13]. 

### 1.2. Emerging PDAC Molecular Subtypes 

There are four broad molecular subtypes of PDAC: a ‘classical’ and ‘basal’ subtype, and the emerging ‘stromal’ and ‘immunogenic’ subtypes [9,14,15,16]. Recent studies have begun to correlate histological differentiation grade with PDAC molecular subtypes to improve the prediction of patient outcomes, with a basal signature enriched in the high grade tumors, whereas a classical signature is enriched in the low grade tumors in patients, patient derived xenografts, and murine tumors [12,17,18] (Figure 1A).

#### 1.2.1. Classical Subtype

The ‘classical’ PDAC signature (Figure 1B) is the most common subtype, regardless of clinical stage [16], and is characterized by the upregulation of transcription factors associated with pancreatic lineage differentiation, including *HNF1A*, *GATA4*, *GATA6*, and *NKX2-2* [15,16,19,20]. Similarly, the ‘pancreatic progenitor’ subtype is defined by an increase in progenitor signature expression, including *PDX1*, *MNX1*, *FOXA2*, *FOXA3,* and *HES1* [14]. Recently, the classical subtype was divided into ‘classical-A’ and ‘classical-B’, with the former having a lower frequency of intact *SMAD4* [16]. A new ’hybrid‘ classical subtype has also been described, which had previously been inconsistently classified within other subtypes due to the presence of overlapping expression signatures [14,15,16,19,20]. 

The ‘exocrine-like’ [17] or the ‘aberrantly differentiated endocrine (ADEX)’ [14] subtypes were previously captured under the ‘classical’ subtype [16,19,20] (Figure 1B), and are associated with acinar cell differentiation, endocrine differentiation, and terminally differentiated pancreatic tissue [14,15]. ADEX and exocrine-like tumor samples have been associated with low tumor cellularity, suggesting that the stromal and normal cells in the pancreas may be the main contributors to the definition of these subtypes [19,21]. However, the exocrine-like subtype has also been described for patient derived xenografts and pancreatic cancer cell lines, suggesting that the signatures are likely derived from tumor cells [22,23,24]. 

#### 1.2.2. Basal Subtype

The majority of metastatic PDAC tumors are of the ‘basal’ subtype [15,20]. This subtype is also known as ‘quasi-mesenchymal’ [15], or ‘squamous’ [14], (Figure 1B) characterized by the expression of laminins and keratins, and enriched for genes associated with epithelial–mesenchymal transition (EMT) and TGF-β signaling [14,16,19,20]. Recently, the basal-like subtype was subdivided into ‘basal-like-A’ and ‘basal-like-B’ [16], with ‘basal-like-B’ described as more aggressive, while ‘basal-like-A’ was considered more chemoresistant [16].

#### 1.2.3. Stromal Subtype

The ‘stromal’ subtype can be subdivided into ‘normal’ stroma or ‘activated’ stroma (Figure 1B), with ‘activated’ stroma associated with a worse survival outcome [20]. Other studies have described a ‘stroma activated’ subtype characterized by a high expression of *ASMA*, *SPARC,* and *FAP*, and a ‘desmoplastic stroma’ subtype, with a high expression of structural and vascularized stromal components [19], both of which were negative prognostic factors [19]. 

Both human PDAC tissue and a murine model of PDAC also revealed the existence of three cancer-associated fibroblast (CAF) subtypes [25,26,27]: inflammatory CAFs (iCAFs), activated by paracrine factors from cancer cells; myofibroblastic CAFs (myCAFs), which are dependent on juxtracrine interactions with cancer cells; and antigen presenting CAFs (apCAFs), which express genes from the antigen-presenting major histocompatibility complex (MHC) class II family [25,26,27]. 

#### 1.2.4. Immunogenic Subtype

The ‘immunogenic subtype’ (Figure 1B) is defined by an immune signature [14]. The ‘classical’ subtype can also be subdivided into ‘pure classical’ and ‘immune classical’. The ‘immune classical’ subtype reflects an increased infiltration of B cells, T cells, and natural killer (NK) cells, lower levels of inflammatory components and activated stroma [19], and is associated with longer survival compared to the ‘stroma-activated’ subtype [19]. The ‘basal-like’ subtypes are generally predicted as having an activated stroma, whereas the subtypes with high immune infiltrate (desmoplastic and immune classical) are predicted to have normal stroma [19].

### 1.3. Current Treatment Options

PDAC often presents with nonspecific symptoms and, at present, there are no reliable biomarkers for routine screening. Consequently, many patients have advanced stage disease at initial diagnosis [28] and only 15–20% of patients are eligible for surgical resection [4], the only potentially curative treatment. Unfortunately, even after surgical resection, 5-year survival rates for pancreatic cancer patients range between 27–39%, due to the lack of effective treatments [29]. Recurrence occurs in 80–85% of patients despite a curative intent resection, with patients identified to have residual postoperative circulating tumor DNA at the greatest risk [30]. Results from the PRODIGE-24 and ESPAC-4 randomized controlled studies demonstrate that the use of adjuvant modified FOLFIRINOX (a combined regimen of 5-FU, leucovorin, irinotecan, and oxaliplatin; 8.8 months improvement in median OS) or combined gemcitabine with capecitabine (2.5 months improvement in median OS), both improve OS when compared with gemcitabine alone [31,32].

In the locally advanced or metastatic disease setting, chemotherapy remains the cornerstone of treatment. Combination regimens including gemcitabine with nab-paclitaxel [33,34] or FOLFIRINOX [35] have demonstrated small incremental improvements in median OS from 8.5 to 11 months [35,36], and remain the first line treatment options [37]. In patients that remain fit for second line therapy at disease progression, median OS from diagnosis is approximately 14.2 months [38], with treatment typically consisting of one of the options not used in the first line. Clearly, there is an urgent need to step outside the current treatment paradigm and investigate new opportunities to improve outcomes for patients with PDAC.

Only a small number of targeted therapies have been developed for PDAC, and for those that have entered clinical trials, the vast majority have failed to improve outcomes [39]. The use of an inhibitor of epidermal growth factor receptor (EGFR) signaling, erlotinib, in combination with gemcitabine was approved by the FDA in 2007; however, the clinical benefit was modest (0.33 months improvement in median OS), and its efficacy likely limited to *KRAS* wild-type patients, hence its use has not been widely accepted in routine practice [40,41]. More recently, the use of Olaparib, a poly-adenosine diphosphate [ADP]–ribose polymerase (PARP) inhibitor, has been formally recommended for patients with germline *BRCA* mutated metastatic pancreatic cancer, who have not progressed following first line platinum-based therapy, based on results from the POLO trial [42]. This study demonstrated a significant improvement in median progression-free survival (PFS) from 3.8 months up to 7.4 months [43]. However, it should be noted that the use of PARP inhibitors only applies to the small subgroup of approximately 7% of PDAC patients with *BRCA* mutations, and chemotherapy was not included in the control group [43]. Furthermore, at interim analysis, there was no significant difference in OS when PARP was added to the treatment [43]. However, it is possible that the OS results may have been confounded by the use of subsequent therapies, including nine patients from the placebo group who were allowed to cross over to receive PARP therapy at progression. 

Other recent clinical trials using targeted agents in combination with gemcitabine include bevacizumab (VEGF-A inhibitor) and sorafenib (multi-kinase inhibitor); again, these drugs did not improve OS [44,45,46]. Unfortunately, there were no predictive markers to assist with the stratification of patients. There is an urgent need for a better understanding of the patients who are best suited to each targeted treatment.

## 2. Preclinical Models of Pancreatic Ductal Adenocarcinoma

Preclinical models have enabled an improved understanding of the genetic and molecular drivers of PDAC, the composition of the tumor microenvironment and the development and testing of new therapeutic opportunities. Traditionally, preclinical models included genetically engineered mouse models (GEMM) or immortalized human PDAC cell lines; however, new patient derived 3D models [47,48,49,50,51,52,53] are gaining increasing traction for use in drug discovery, and are reshaping the approach to personalized medicine.

### 2.1. Genetically Engineered Mouse Models

The progression of PanINs to PDAC can be mimicked by the pancreas-specific expression of tumor associated genes in GEMMs, the most commonly used of which is the lox-stop-lox (LSL)-*Kras*^G12D/+^; LSL-*Trp53*^R172H/+^; *Pdx-1*-Cre (KPC) mouse [54]. Pancreas specific gene expression is achieved through targeting pancreatic and duodenal homeobox 1 (*Pdx-1*-Cre, expressed at E8.5 [55]). *Pdx-1* driven cre recombinase permits the constitutive expression of a transition mutation that activates the Ras effector pathway in ductal epithelial cells (LSL-*Kras*^G12D^ [54]), in addition to the concurrent expression of a point mutation functionally equivalent to a dominant negative mutation in the tumor suppressor p53 (LSL-*Trp53*^R172H^ [54]). Sporadic tumors are evident in KPC mice by four months of age [56], with metastases to the liver, lung and peritoneum, similar to human PDAC, present in 80% of the KPC mice [56]. 

Additional variations of the KPC model include the use of a LSL-RosaYFP reporter allele (known as the KPC-Y model), enabling the detection and isolation of YFP+ cells, and revealing that metastasis is an early event in pancreas tumorigenesis [57,58,59]. Similarly, the use of the ‘Confetti’ allele (known as the KPC-X model), which permits the stochastic expression of one of four fluorescent colors (cyan, green, red, and yellow), has demonstrated the clonal diversity present in PDAC metastasis [60]. The analysis of tumors from these KPC-X mice demonstrated polyclonal populations of cells in the primary tumor, which were able to seed distant metastases at sites such as the peritoneum, liver, and lung. While peritoneal metastases were mainly comprised of polyclonal populations (up to 80%), the liver and lung metastases, in contrast, usually displayed outgrowths of single clones, with a smaller percentage (11–14%) of tumors at these sites displaying polyclonality [60]. The ability to track metastasis enables a better understanding of the EMT processes, including how modifying cell adhesion proteins, including N-cadherin and E-cadherin [61,62], or EMT transcription factors, such as ZEB1 and SNAI2 [63,64], may contribute to metastasis. 

KPC mice have enabled the understanding of the contributions of additional genetic aberrations to PDAC, including *CDKN2A* or *SMAD4* loss (genetic features commonly observed in patients [5]), which results in an increased frequency of metastasis [65,66,67]. Deletion or mutation in genes such as *Gli1,* which is involved in hedgehog signaling pathways [68] and familial pancreatic cancer genetic signatures, including *Brca2* or *Lkb1* [69,70], have also been explored in KPC mice. 

The sporadic formation of PDAC in KPC mice occurs in parallel with the formation of a highly desmoplastic tumor environment, which mimics the human PDAC tumor microenvironment [25,26,27,54,71,72]. Macrophage and myeloid cells dominate the immune microenvironment in both the primary and metastatic tumors of KPC mice, similar to the human disease [71]. Myeloid cells promote tumor cell migration, leading to tumor cell invasion in local tissues and metastasis [73]. In contrast to myeloid cells, regulatory T cells are most prevalent in PanIN lesions and implicated as a key promoter of PDAC progression due to their ability to release immuno-suppressive cytokines that hinder effector T cell activity [74]. Depleting regulatory T cells was shown to prolong the survival of KPC mice [74,75]. These features have enabled opportunities to enhance immune mediated tumor destruction, through the genetic or pharmacological inhibition of numerous immune checkpoint inhibitors [76]. 

KPC mice model both the ‘classical’ and ‘basal-like’ PDAC molecular subtypes [14,16,18]. KPC tumors contain different stromal populations, including the iCAF and myCAF subtypes found in human PDAC [25,26,27], thus permitting a better understanding of how stromal modulators may promote altered response to chemotherapy or targeted therapies [77,78,79]. Similarly, KPC mice have been used to demonstrate that depleting stroma cells in PDAC tumors can improve responses to immunotherapy [72]. Using KPC mice, it was discovered that CAFs produce chemokines, such as CXCL12, that can promote an immunosuppressive environment, preventing the infiltration of cytotoxic T cells to the tumor [72].

#### GEMM for the Generation of Organoids

Organoid culture systems, three-dimensional (3D) in vitro models of tissue systems, have been generated from mouse embryonic pancreatic stem cells to study pancreas development [80]. Embryonic pancreatic organoids proliferate and form branchlike structures *ex vivo*, mimicking normal ductal formation during development [80]. Organoids have also been generated from pluripotent stem cells originating from neonatal or adult pancreas tissue, with the organoid structure, cell types and functions resembling the in vivo tissue from which the organoids were derived [81,82].

Murine organoids have facilitated studies into the processes underlying the development of PDAC, as they can be generated from different stages of disease by using tissue from the KPC GEMM [83]. However, this model represents specific genetic mutations, and is not representative of all genomic and molecular patient subtypes. Murine organoids have been used to study how *KRAS^G12D^* mutations affect tumor proliferation [84]. Murine pancreatic ductal organoids with *KRAS^G12D^* are able to bypass cellular senescence and grow in syngeneic allograft models, while wild type organoids fail to engraft [84]. Analysis of the growth and differentiation of matched murine organoids from primary and metastatic tumors has improved our understanding of how epigenetic changes facilitate a more aggressive state in metastatic organoids [85]. This led to the discovery that *FOXA1* drives enhancer reprogramming during the progression of PDAC [85]. This would not have been ethically feasible in human organoid systems, where it is not safe to obtain biopsies of both the primary and metastatic tumors, which is only possible following rapid autopsy.

Organoids also provide an opportunity to identify the novel biomarkers of PDAC progression, which would potentially allow earlier detection of the disease [83]. For example, the glycan carbohydrate antigen 19-9 (CA19-9), which is often elevated in patient serum and is used as a marker to monitor disease progression, has also been shown to be expressed in patient derived organoids (PDOs) [86]. To study the involvement of CA19-9 in pancreatitis, organoids were generated from transgenic mice that expressed CA19-9 and retained the ability to express CA19-9 [87]. Conditioned media collected from the CA19-9 expressing organoids stimulated EGFR phosphorylation, suggesting a role for CA19-9 in EGFR mediated chronic pancreatitis [87]. 

Organoids maintain the in vivo characteristics of pre-neoplastic cells and are able to progress into invasive tumors following orthotopic transplantation in syngeneic hosts [24,83]. Murine organoids generated from either preinvasive or neoplastic pancreases have been injected into diabetic mice and have shown, in vivo, that the diabetic microenvironment promotes PDAC progression [88]. Orthotopic syngeneic transplant models assist with studying the role of immune cell infiltration as lesions progress from preinvasive to metastatic disease [24], which is not possible in immune incompetent patient derived xenografts. However, the site of organoid engraftment is an important consideration, as a study comparing injecting organoids into the pancreatic interstitial space versus the major pancreatic duct found that the ‘squamous’ signature is enriched in the interstitial engraftment, whereas the ductal engraftment enriched the ‘progenitor’ and ‘classical’ signatures [89].

### 2.2. Patient Derived Models

#### 2.2.1. Monolayer Cell Culture

There are 11 well established two-dimensional (2D) human PDAC monolayer cell lines that have commonly been used to understand the genetic landscape of PDAC and investigate new therapeutic opportunities, in vitro [90]. These cell lines were derived from five female and six male Caucasian patients, ranging from 26 to 65 years of age [90]. Of these, five were generated from primary tumors [91,92,93,94,95,96], with the remaining generated from liver or lymph node metastases or ascites [96,97,98,99,100,101]. All of these cell lines have mutations in the four most commonly mutated genes (*KRAS, TP53, CDKN2A,* and *SMAD4*), with the majority of these cell lines harboring either a G12D or G12V mutation in *KRAS*, with the exception of BxPC-3 which is wild-type for *KRAS* [90]. However, an emerging limitation is that the genetic profile and gene expression of 2D PDAC cell lines are significantly different to the original patient tumor [102], which may be due to the 2D cell lines representing clonal populations that grow as monolayers [103,104]. Transcriptomics analysis from 22 tumor types of primary tumors from The Cancer Genome Atlas (TCGA) and cell lines from the Broad Institute Cancer Cell Line Encyclopedia (CCLE) found that, while the molecular profile of primary tumors generally correlated with their corresponding tumor cell line, the primary tumors showed the enrichment of gene sets related to immune cell signaling (i.e., the microenvironment), while the cell lines showed the enrichment of gene sets involved in cell-cycle progression [102].

Since the growth of monolayer cultures does not recapitulate the complex 3D architecture of the original tumors in vivo [105], orthotopic or subcutaneous xenografts of cell lines into immunocompromised mice are often utilized in parallel [106]. However, they do not necessarily recapitulate the histological phenotype of a PDAC tumor, and the weakened immune system in xenograft models impacts the extent of the accurate representation of the tumor microenvironment, which may contribute to the poor correlation between the responses of these cell line xenografts to therapeutics and clinical trial results [107,108]_._

#### 2.2.2. Cell Line Derived Spheroids

Initial attempts to culture fresh pancreas tissue *ex vivo* ranged from maintenance of a whole cat pancreas in culture using a perfusion pump [109], to culturing vibratome-sectioned murine pancreas tissue slices [110], with each technique challenged by the inability to maintain viable cells in culture for long periods of time, and not being able to successfully passage and biobank the cells [111]. More recent efforts have focused on methods to enzymatically dissociate normal and neoplastic pancreas tissue, and propagate the epithelial cells in 3D culture systems [112]. One of the first in vitro 3D culture methods was the generation of ’spheroids’ [113]. 

The Capan-1 cell line was the first spheroid culture generated for PDAC [113], which, when grown in suspension, self-organized into hollow spheres composed of a single layer of polarized epithelial cells, termed ’spheroids’ [113]. Since then, 3D cultures have been successfully generated from the PANC-1 [114], AsPC-1 [115], MiaPaCa-2 [115], Capan-2 [116], and BxPc-3 [116] cell lines, and, although not all cell lines formed spheroids, 3D cultures derived from cell lines were collectively termed as spheroids [114,115,116,117]. However, one of the major setbacks for spheroid cultures generated from 2D cell lines is that the differentiation and selection of subclones may have already occurred while in monolayer culture; hence, a change in the morphology, function, and biochemistry of the cell line would potentially lead to a loss of phenotypic diversity and, thus, may not fully recapitulate the complex environment of the original tissue or tumor [118].

Spheroids have shown similar expression signatures to cells grown in xenograft models, including the upregulation of drug resistance related molecules, cytokeratin, and extracellular matrix (ECM) [116,119,120,121]. Drug sensitivity testing also highlighted that spheroid cultures had higher drug resistance compared to the corresponding monolayer cultures due to the presence of ECM components in the matrix, which recapitulates the chemoresistant nature of PDAC [119]. Compared to 2D cell cultures, spheroid cultures may be more appropriate for the profiling of drug responses in patients [119,122], due to the upregulation of genes that match xenograft expression profiles, with either model more cost effective than organoid systems. 

#### 2.2.3. Patient Derived Xenografts

Approximately 70% of PDACs arise within the head or neck of the pancreas, while roughly 13% and 17% occur in the body and tail, respectively [123,124]. The most common site of distant metastases is the liver, followed by peritoneum and lung [56]. Biopsy or the surgical removal of tissue from each of these sites has enabled the development of a diverse range of PDAC patient derived xenografts (PDXs). Seven PDAC PDX libraries have been established in the last 10 years, with the largest to date establishing PDXs from 57 patients, with an average engraftment success rate of 48% [125,126,127,128,129,130,131]. In general, PDXs are established from the implantation of tumor tissue subcutaneously, or occasionally orthotopically, into immunocompromised mice [132]. The desmoplastic tumor microenvironment is replaced and maintained by the murine stroma [131,133], recapitulating the histopathological features of the patient tumor [131,133]. PDAC PDXs have been shown to retain the mutation signatures of the corresponding tissue, even after multiple passages [131,133]. 

A pilot clinical study in patients with advanced pancreatic cancer generated PDXs demonstrated significant correlation between the drug responses of the PDXs and patients’ therapeutic response, highlighting the potential of PDXs to guide patient treatment selection [134]. Several other studies have used PDAC PDXs to identify potential biomarkers for pancreatic cancer, and investigated the efficacy of targeted therapeutics in combination with standard-of-care chemotherapy [135,136,137]. Despite the obvious attraction of treatment selection informed by PDX drug sensitivity, the clinical uptake of PDXs to inform personalized medicine is hindered by the cost, the reliability, the complexity and the substantial time (often > 6 months) required to establish PDX models [138,139].

#### 2.2.4. Patient Derived PDAC Organoids

3D organoid systems for the pancreas were initially generated from mouse embryonic pancreatic stem cells to study pancreas development [80]. The protocols established to understand normal pancreas development in mice were extended to study human pancreas biology and PDAC development [140]. The differentiation from human embryonic stem cells into pancreatic ductal organoids in culture, followed by transduction with lentiviral vectors for *KRAS^G12V^* and *TP53^R175H^*, enabled the study of pancreatic tumorigenesis [140]. In comparison to normal human adult pancreas cells, the tumor organoids were found to express higher levels of progenitor markers, including *PDX1*, *SOX9*, and *NKX6.1*, and to have a lower expression of differentiated acinar, ductal and islet markers, suggesting that the tumor organoids were mainly ductal progenitor cells in origin [140]. 

Organoid culture conditions are more complex than monolayer or 2D cultures, and are continually evolving. Combined removal of R-spondin-1, EGF, or phorbol myristate acetate reduces murine ductal cell organoid culture efficiency, although removal of these components individually did not appear to affect organoid formation [80]. Fibroblast growth factor 10 (FGF10) has been shown to reduce acinar dedifferentiation in murine organoids [80]. As a result of these, and other studies, murine organoid protocols are now based on a combination of niche factors, including R-spondin-1, EGF, FGF10, Noggin, and nicotinamide, to generate cystic sphere-shaped organoids growing in Matrigel from adult murine pancreatic ductal cells [112]. Flow cytometry to detect the markers of different cell subtypes has determined that these media conditions only support the propagation of ductal epithelial cells, and not acinar or islet cells [112]. 

**Figure 2:**
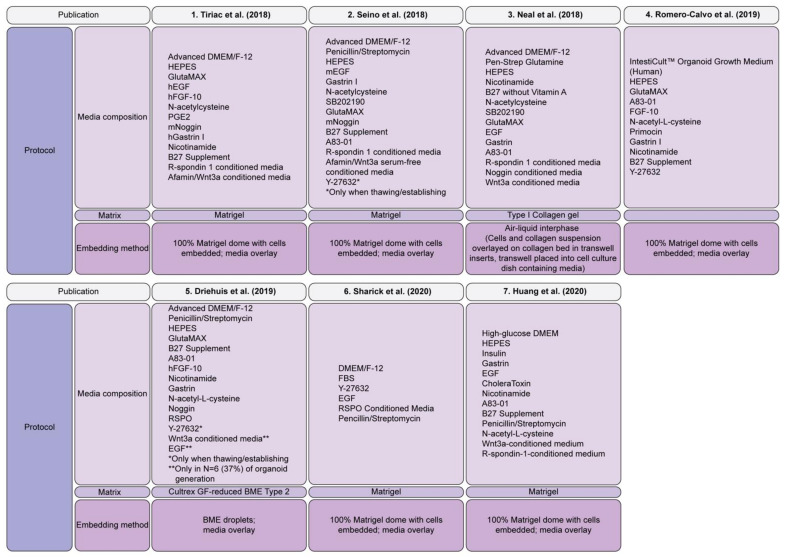
PDAC organoid culture methods. Summary of the protocols utilized by the seven PDAC organoid biobanks described to date [47,48,49,50,51,52,53]. Abbreviations: Media Composition: DMEM: Dulbecco’s Modified Eagle Medium; hEGF, human recombinant epidermal growth factor; mEGF, mouse recombinant epidermal growth factor; hFGF-10, human recombinant fibroblast growth factor-10; PGE-2, prostaglandin E2; mNoggin, mouse recombinant Noggin, hGastrin I, human recombinant Gastrin I.

In recent years, a focus on the improvement of murine protocols for the generation of patient derived PDAC organoids has included EGF, R-spondin, Noggin, Gastrin I, Nutlin3, A83-01 (an ALK5/7/4 inhibitor), nicotinamide, N-acetylcysteine, B27 supplement, Wnt3a, and Primocin in the culture media (Figure 2) [48,83], which has been extensively reviewed elsewhere [141]. In the normal human adult pancreas, Wnt signaling is inactive and is only robustly activated upon injury, which is associated with regenerating pancreatic ducts [142]. Thus, the organoids generated with Wnt ligands in the culture media are likely to be a model for the regenerative state [142]. KRAS is a downstream effector protein in the EGF signaling pathway which regulates cell growth [143], whereas Noggin is an antagonist for bone morphogenic protein (BMP) 4, which belongs to the TGFβ superfamily [144] and is critical for the normal growth and development of pancreatic tissue. EGF withdrawal from the organoid media allows the selection of *KRAS* mutant organoids, as an oncogenic *KRAS* mutation causes the constitutive activation of the downstream EGF pathway, resulting in sustained organoid growth independent of EGF stimulation. The effects of genetic alterations on the growth factor requirements of the organoid cultures have also been observed in gastric cancer [145], highlighting the importance of understanding the underlying genetic events to determine niche factor requirements for the generation of patient derived organoids from the spectrum of PDAC molecular phenotypes. 

Initially, normal human pancreatic organoids could only be cultured for 20 passages before proliferation ceased [83,112]. This was associated with serum-containing Wnt conditioned media, as the serum was found to promote differentiation in culture, which also triggers senescence in normal pancreatic organoids [83,112]. Using serum free Afamin-stabilized Wnt3a conditioned media, normal pancreatic organoid cultures were able to grow for an additional five months, compared to previous protocols [48,146]. In contrast, human PDAC organoids have been shown to passage indefinitely, although they can be plagued by the outgrowth of normal tissue organoids [83]. This is difficult to monitor, even by using flow cytometry, as both normal and PDAC ductal epithelial cells have similar epithelial markers. PDAC organoids can also be selected from normal tissue organoids based on other niche factor requirements, such as the addition of Nutlin3 (an MDM2 inhibitor/p53 inducer), A83-01 treatment and TGFβ1 removal, or by Noggin removal and BMP4 treatment [48].

#### Patient Derived Organoid Biobanks

**Figure 3:**
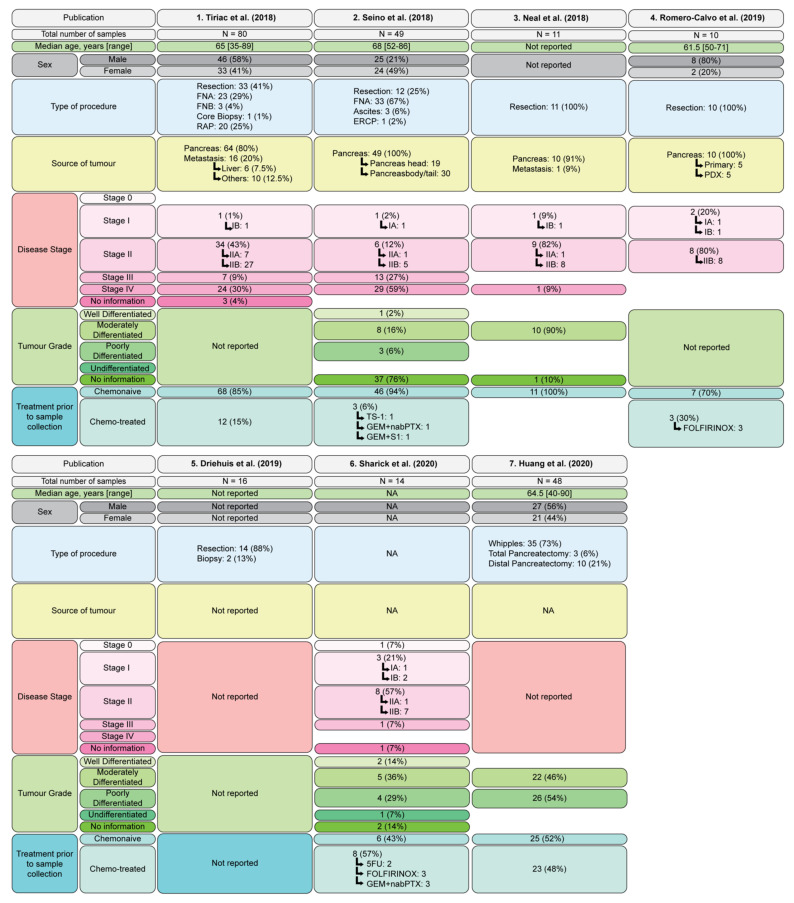
Features of PDAC organoid biobanks. Summary of the seven PDAC organoid biobanks described to date [47,48,49,50,51,52,53], including total number of samples, median age, sex, type of procedure, source of tumor, disease stage (according to AJCC Cancer Staging Manual, Eight Edition [11]) and treatment prior to sample collection. Abbreviations: Source of tumor: PDX, patient derived xenograft. Treatment prior to sample collection: chemotreated: GEM, gemcitabine; nabPTX, nab-Paclitaxel; FOLFIRINOX, fluororacil (5FU)+folinic acid/leucovirin+irinotecan+oxaliplatin.

Seven PDAC PDO biobanks have been described to date (Figure 2 and Figure 3). The first, and most comprehensive, includes 114 PDO generated from 101 patients (with a success rate of 72% for endoscopic ultrasound guided fine needle aspiration (EUS-FNA) samples and 78% for tumor resections) [47]. The histopathological grades of the original tumors used for PDO generation were not reported (Figure 3). Sanger sequencing for *KRAS* or whole exome sequencing on 88 PDOs confirmed 69 (78%) of the PDOs were derived from the tumor cells, as they harbored genetic alterations consistent with PDAC; however, 19 (22%) of the PDOs arose from the outgrowth of normal cells [47], ultimately meaning that the success of growing PDAC tumor organoids is lower than the reported success of PDO generation.

Transcriptomics analysis of 44 PDOs in the first biobank [47] revealed that 31/44 (70%) had a ‘classical’ PDAC signature, whereas 13/44 (30%) had a ‘basal-like’ signature. These PDOs were also independently classified into two clusters: cluster 1 was enriched for TGFβ signalling and EMT, and cluster 2 was enriched for xenobiotic metabolism, fatty acid metabolism and oxidative phosphorylation [47]. There was no overlap between the genes defining clusters 1 and 2, and the signatures of ‘classical’ and ‘basal-like’ subtypes; however, there was a correlation between the classifications, with 83% of ‘basal-like’ PDOs falling under cluster 1, and 93% of ‘classical’ PDOs under cluster 2, suggesting correlation between gene expression signatures and affected molecular pathways [47]. 

The second biobank includes 49 PDOs generated from surgical resections (12/49), FNA (33/49), and ascites specimens (3/49) [48]. In this biobank, 37/49 samples had no histological information provided, while 1/49 (2%) of the PDOs were generated from a well differentiated tumor, whereas 8/49 (16%) and 3/49 (6%) were derived from moderately and poorly differentiated tumors, respectively (Figure 3). Of these, EGF-based selection and niche-based selection confirmed that 39 were PDAC organoids (80%), and the remaining 10 organoids had arisen from normal cell outgrowth (20%), which was confirmed by whole exome sequencing. Each of the normal cell organoids were found to be dependent on Wnt signaling; however, it was observed that the PDAC organoids had three Wnt niche subtypes, including Wnt dependent, Wnt independent and Wnt producing subtypes, unrelated to mutations in the Wnt signaling pathway [48]. 

The third biobank successfully generated 11 PDAC PDOs, with a success rate of 65% (11/17) [49]. Of these PDO, 10 of 11 (91%) were generated from moderately differentiated tumors, with no information provided for the remaining PDO. Target exome sequencing and copy number variance analysis confirmed that all 11 of the PDOs were PDAC and exhibited the expected mutations, including recurrent mutation in *KRAS* and *TP53* [49]. 

The fourth biobank was generated from five PDXs in addition to five PDOs [50]. Tumor samples were obtained from pancreatic resections and confirmed to be tumor derived based on pathological assessment [50]. Although the histopathological grades of each of the tumors used for PDO generation were not reported, histological comparison showed a high degree of correlation between the primary tumors and PDO or xenograft-derived organoids. Single cell RNA sequencing was also performed on one organoid in this PDO biobank, which revealed transcriptionally distinct subpopulations in PDOs that were characterized into three clusters, with 97.2% of the cells from a single clone, indicating that PDOs are primarily clonal populations derived from the primary tumor [50]. 

The fifth biobank includes 83 patient samples, from which 52 PDOs (63%, 52/83) were generated successfully. Of these PDOs, whole genome sequencing revealed that 30 PDOs were tumor organoids (57%, 30/52), suggesting that normal outgrowth may impact actual tumor PDAC organoid success rates. Histopathologic analysis of the parental tumor tissues confirmed that out of these 30 PDOs, 1 was an adenosquamous, 1 was an acinar cell carcinoma, 2 were cholangiocarcinomas, 1 was a duodenal carcinoma, 1 was an ampulla of Vater adenocarcinoma, 14 were PDAC, 2 were derived from IPMN and 9 of the organoids did not have a matching pathology report. This means there were 16 validated PDAC PDOs in this biobank [51].

A sixth biobank includes 14 PDOs successfully generated from 22 patient tumor samples (64%, 14/22) [52]. Out of the 14 established PDOs, 10 were derived from PDAC and 1 was an ampullary adenocarcinoma. Of the patient samples, 57% (8/14) had received neoadjuvant treatment prior to resection and collection of the tissue; however, this did not appear to have an impact on organoid formation, as success rate for pre and nontreated samples were similar (67% and 60%, respectively). High depth targeted gene sequencing was only performed on one PDO, limiting the knowledge of the mutational profiles of all the PDOs in this biobank, and validation that they are tumor in origin and not organoids derived from normal cell outgrowth [52].

The seventh PDAC PDO biobank, consisting of PDOs isolated from 48 primary human PDAC samples, was established (success rate was not reported) [53]. The first 25 of these PDAC samples were subjected to immediate invasion analysis, whereas the remaining 23 samples were generated for additional analyses, including immunofluorescence, lentiviral transduction, and pharmacological manipulation [53]. Deep targeted next generation sequencing analysis was only performed on the first 25 primary PDAC samples to determine somatic mutations; however, sequencing was not performed on matched PDOs. Other studies have shown that the mutational profile of PDOs may not be representative of the patient’s tumor, with normal outgrowth a major limitation in PDO biobanking [47,48]. 

## 3. Organoids, an Emerging Model System to Understand PDAC Biology

### 3.1. Organoids Permit a Deeper Understanding of the Molecular Features of PDAC 

To date, most of the large-scale genomic and transcriptomic studies in PDAC have been performed on surgical samples [14]. More recently, studies have focused on the genomic analysis of matched PDOs, as they overcome the problem of immune and stromal contamination in tissue samples and serve as an enriched source of tumor cells. Three independent studies have shown that PDAC PDOs had similar genomic signatures to the original patient tumors across multiple passages [47,48,50]. However, despite similarity in the genetic composition of PDOs and their corresponding primary tumor, there has been some differences in the overall mutational load reported in these studies [147]. While a high percentage of the somatic mutations detected in the primary tumor were also found in the organoid cultures (average 97%), concordance between the two was more variable (range 8–96%, 6/13 samples >80%) as additional somatic mutations were detected in organoids [47]. Similarly, for five patient tumor and organoid pairs analyzed for structural variation events (inversion, duplication, inversion or translocation), four demonstrated concordance scores < 0.55 [147]. Additionally, it has been observed that the variant allele frequency was generally higher in organoids compared to the tumor of origin [50]. Where differences between the organoid and original tumor are apparent, this may be due to the presence of large immune and stromal contamination in the tumor.

The transcriptome of the organoids has also been shown to be comparable to the primary tumor (R^2^: 0.66); however, it should be noted that this was analysis of only one patient, and, therefore, correlation may differ between PDAC subtypes [50]. A comparison of differentially expressed genes from a primary tumor, 2D cell line, PDX, and an organoid derived from one patient found that the majority of genes were expressed similarly between the four different models [50]. This suggests that organoids may retain their ‘classical’ and ‘basal-like’ signatures and may serve as a model system to study different subtypes of PDAC [47,48,50].

### 3.2. Organoids Provide a Unique Insight into Metastatic Processes

Organoid based drug sensitivity studies have revealed that two tumors derived from a single PDAC patient can respond differently to chemotherapy [47]. For example, the generation of PDOs from different metastatic sites of the same patient has demonstrated different sensitivity to 5-FU, despite the organoids harboring a similar DNA mutational profile [47]. This may be due to the differences observed in copy number alteration between these organoids, or may suggest the presence of nongenetic or epigenetic differences between tumor cells at different sites of metastasis, and potentially the presence of clonal tumor cell populations which may contribute to the heterogeneity in therapeutic profiles within the same patient [47].

Organoids have also been used extensively to understand EMT, which is a mechanism used by the tumor cells to metastasize and possibly to develop drug resistance [148]. Culturing organoids in a transparent 3D matrix allows for the observation of cell invasion, providing valuable insights into the dynamics of cancer cells [53,149,150]. Studies such as these have revealed that SMAD4, which is commonly mutated in PDAC, promotes EMT [53]. Similarly, the contribution of TGFβ to the EMT process in PDAC has been studied using human PDAC organoids [53,151,152]. Murine PDAC organoid studies have revealed that the loss of E-cadherin results in the formation of poorly differentiated tumors in orthotopic models [18,57,62]. 

### 3.3. Modelling the Stromal Tumor Microenvironment Using Organoid Cultures

PDAC is characterized by a desmoplastic stroma, comprised of stiff ECM, CAFs, and immune cells that account for more than 80% of the tumor mass [153,154]. The secreted ECM includes collagens, fibronectin, and laminin, which provide a shield protecting cancer cells from chemotherapeutics, while simultaneously creating a network through which cells can migrate and invade [155,156,157]. With this complex ECM in vivo, matrix selection for the in vitro propagation of organoid cultures is important [158]. The stiffness and degradability of the matrices impact cell proliferation differentiation within the organoids, which can change growth and, thus, the drug responses of the organoids and limit drug penetration [148,159,160,161,162,163,164].

Driven by the recognized importance of immune and stromal cells to tumor growth and drug responses, the use of cocultures is gaining momentum as a research tool. The coculture of fibroblasts and tumor cells was first utilized in cell-line derived spheroids [161,162]. It was found that coculture with a human pancreatic stellate cell line (HPaSteC, ScienCell) resulted in an increase in tumor cell proliferation, cell motility and upregulation of TGFβ, and connective tissue growth factors in the spheroids [161]. Similarly, the coculturing of spheroids, with primary PSCs isolated from human PDAC tumors, also induced EMT [162]. Importantly, spheroid coculture with PSCs also resulted in increased chemoresistance to drugs such as gemcitabine and oxaliplatin [161,162,163,164]. 

In early cultures of PDO, fibroblasts were visible in culture and attached on the well surface, allowing the isolation and expansion of the fibroblasts as 2D cultures [49,163]. Multiple methods to facilitate the coculture of stromal fibroblasts with organoids have now been described, including the direct embedding of cocultures in Matrigel, air–liquid interfaces (ALI), or Transwell^®^ coculture systems [49,84,165]. The Transwell^®^ system permitted the characterization of paracrine interactions between murine organoids and CAFs, including the increased secretion of interleukin-6 and leukaemia inhibitory factor by CAFs, which have been reported to promote PDAC progression [25,166,167,168,169]. Studies such as these have shown that CAFs secrete factors that contribute to the loss of basement membranes and ductal structures in organoids, facilitating invasion into surrounding matrices [170]. Both the in vitro coculture experiments and the transplantation of organoids with CAFs into mice demonstrated that the physical contact of CAFs allowed non-Wnt producing organoids to overcome the lack of Wnt supplied in the media [48]. 

It should be noted that both murine and human PDAC have three CAF subtypes: iCAFs, myCAFs and apCAFs [25,26,27,28]. The ability to maintain the different CAF subtypes *ex vivo* has not yet been explored. Moreover, these different fibroblast populations reside in different regions of a primary tumor, making isolation of specific CAF subtypes challenging [25,26]. 

### 3.4. Modelling the Immune Microenvironment in Organoid Cultures

The general immune cell populations present in, and around, PDAC include cytotoxic T cells, regulatory T cells, NK cells, myeloid-derived suppressor cells (MDSCs) and macrophages [171,172]. A PDAC organoid coculture model, utilizing both stromal (CAFs) and immune components (T lymphocytes), has now been reported, and demonstrates that the inclusion of these cells increased organoid resistance to gemcitabine [163]. However, T cells growing in the organoid growth media had limited viability and demonstrated a less activated phenotype, highlighting that the organoid media conditions may not be compatible with the maintenance and viability of immune cells [163]. Other studies have described the coculture of monocytes, CAFs and organoids, which reportedly increased the secretion of immunosuppressive cytokines, which further inhibited T cell activation and proliferation in vitro [173]. 

The coculture of human and murine PDAC organoids with MDSCs, differentiated from either murine bone marrow or patients’ peripheral blood mononuclear cells, promoted tumor growth and inhibited cytotoxic T cells proliferation [174]. In the presence of MDSCs, PD-L1-expressing organoids were unresponsive to a PD-1 receptor inhibitor, nivolumab [174].

**Figure 4:**
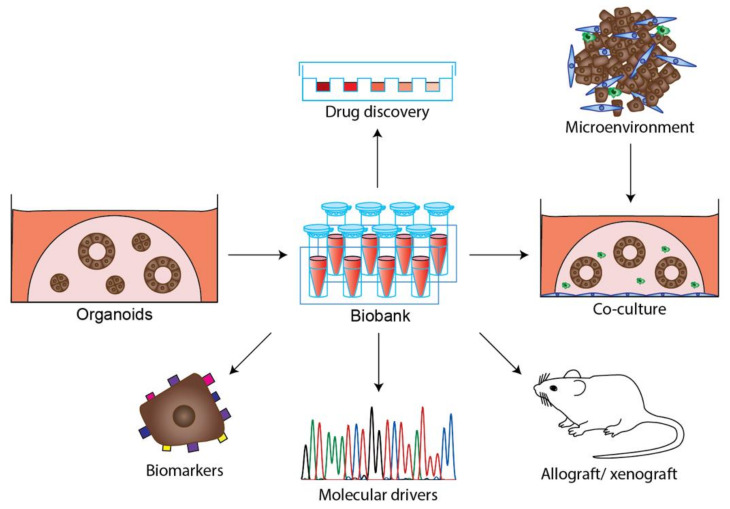
Diverse applications of PDAC tumor organoids. PDAC tumor organoids can be stored as biobanks and utilized to address multiple research questions, including drug discovery, identification of biomarkers and molecular drivers. Organoids are emerging as model that integrate the microenvironment through coculture or transplantation in mice as allograft or xenografts.

Another coculture study has described the modelling of the immune microenvironment in PDAC and other tumor organoid cultures using a modified ALI method [49]. In this system, the viability of immune cells (such as T cells, B cells, NK cells and macrophages) was preserved over the course of several months [49]. Functional tumor-infiltrating lymphocytes were also detected in ALI PDOs derived from melanoma, non-small-cell lung cancer (NSCLC) and renal cell carcinoma, and were clonally expanded, activated and exhibited a cytotoxic response upon blockade of immune checkpoints PD-1/PD-L1 [49]. As coculture protocols develop, new opportunities for immuno-oncology studies with PDAC PDO will emerge (Figure 4). 

## 4. Patient Derived Organoids as a Model to Identify New Treatments for PDAC Patients

Currently, the selection of therapy for individual patients with PDAC is largely informed by clinical trials that have enrolled a wide range of patients, rather than individualized patient management based on data obtained from target panel or aggregate sequencing data. Exceptions include patients with *BRCA* mutations, where PARP inhibitors represent a treatment option [43]. While a consensus regarding the definition of molecular subtypes has yet to be reached for PDAC, molecular phenotyping may further facilitate the stratification of patients for appropriate treatments. Patients with the ‘classical’ PDAC subtype are considered to respond significantly better to first line therapy, when treated with m-FOLFIRINOX, compared to the ‘basal-like’ subtype [175]. This is supported by the observation that with patient stratification based on ‘basal-like’ or ‘classical’ subtypes, the ‘classical’ subtypes lead to a better survival rate [20]. Patients with the ‘exocrine-like’ subtype have been shown to be resistant to tyrosine kinase inhibitors and paclitaxel [22]. It is thought that PDAC patients with an ‘unstable’ subtype, which is suggestive of defects in DNA maintenance, may be more sensitive to DNA-damaging agents [9]. The ‘locally rearranged’ subtype has focal amplifications in potential therapeutic targets, including ERBB2, MET, CDK6, PIK33CA, and PIK3R3 [9]. Those PDAC patients with the ‘unstable’ subtype are suggested to have tumors with a defect in DNA maintenance mechanisms, which should render the tumor susceptible to platinum or PARP inhibitors [9]. While these signatures may guide treatment decisions, they do not guarantee that a patient will respond to the targeted therapy, a challenge that could potentially be overcome by the routine introduction of organoid screening. 

### 4.1. PDAC Organoid Treatment Sensitivity May Predict the Patient Response to Chemotherapy

The ability to appropriately predict a patient’s response to therapy is an important validation of the preclinical utility of organoids. There has been evidence of drug sensitivity in PDO paralleled to patient response in gastrointestinal cancers, such as gastric, rectal and intestinal cancers, suggesting the general utility of organoid cultures as a predictive tool is not cancer-type specific [176,177,178]. 

To date, there has been limited comparison of PDAC PDO response to patient treatment response, with PDAC PDOs in four independent studies subjected to chemosensitivity assays including gemcitabine, paclitaxel, irinotecan, 5-FU, and oxaliplatin screening, which revealed marked interpatient variability to single chemotherapeutic agents [47,50,51,52]. By matching a patient’s clinical response to the drug response of chemonaïve PDOs, it was revealed that the response of PDOs to chemotherapy treatment, including gemcitabine and paclitaxel, largely matched the patient’s responses, noting that discordance was also reported [47,51]. Contaminating normal organoids, or the emergence of dominant clones following passaging, may impact the clinical utility of organoid screening. PDOs subjected to combination therapy, such as FOLFIRINOX and gemcitabine/abraxane, also showed a similar response between the PDX and organoids, and both paralleled the patient’s treatment responses [50]. 

While chemical based drug assays are commonly used, an imaging based drug assay called optical metabolic imaging (OMI) was used to observe the metabolic heterogeneity of PDOs following single and combo drug treatment [52]. The OMI drug assay of organoids was also shown to be able to capture patient’s treatment outcome, which indicates the potential of the OMI of PDOs in predicting a patient’s response to treatment and supporting drug discovery and development [52]. 

### 4.2. Organoids Can Be Used to Identify Potential Targeted Therapies

Organoids also provide an opportunity to explore the efficacy of targeted therapies (Figure 4). For example, PDAC PDOs harboring *ERBB2* amplifications and *EGFR* mutations were sensitive to the tyrosine kinase inhibitor Afatinib, an ERBB directed/mutant EGFR targeting agent, while another PDAC PDO, with an oncogenic *PIK3CA* allele, was sensitive to the mTOR agent Everolimus [47]. In a screen of 24 PDAC PDOs with 76 therapeutic agents (including chemotherapeutics), comparable responses were observed for agents targeting similar molecular pathways or processes [51]. The mutation status of *MAP3K1* and *PIK3R1* was associated with responses to the HER2/EGFR inhibitor lapatinib, in combination with gemcitabine, with increased sensitivity observed in PDOs with abnormal copy numbers of these genes. Similarly, the loss of *MTAP* in PDOs conferred sensitivity to the PRMT5 inhibitor EZP015556. The sensitivity to the AKT inhibitor MK-2206, was decreased for PDOs with copy number alterations in *FGFR1* [51]. Other examples of organoids revealing new therapeutic opportunities include RNA based therapeutics for patients resistant to gemcitabine treatment [179], the inhibition of MAPK interacting protein kinase (MNK) or Enhancer of zeste homolog 2 (EZH2) [140,180], the combined inhibition of EGFR and AKT [181], as well as combined inhibition of ERBB and MEK as potential therapeutic targets to treat patients with PDAC [182].

## 5. Conclusions

PDO is an emerging technology that may significantly alter how we approach personalized medicine in the future. EUS-FNA is a common diagnostic tool for those with suspicious pancreatic masses [183] that is amenable to PDO generation [47,48,184], meaning that organoid based testing could support the neoadjuvant screening of patient drug sensitivity using samples that can be safely obtained at the same time as the diagnostic sample. However, EUS-FNA samples are only collected from a small area of a tumor that is often highly heterogenous, and, thus, may not accurately represent the overall tumor biology. Hence, future studies comparing EUS-FNA and surgical derived organoids would be beneficial to validate the reliability of EUS-FNA derived samples.

The growth of PDOs to screen for drug responses is not dissimilar to the concept behind bacterial swab cultures to identify the appropriate antibiotic treatment for infected patients. However, until culture success rates and the cost of organoid expansion are drastically improved, the clinical utility of PDOs will remain limited. The development of artificial intelligence technologies, to automate screening and report results in a clinically useful manner, is also required. Moreover, for PDO to be truly representative of a patient, all components of the tumor microenvironment and their impact on treatment response may need to be considered. As culture conditions evolve, our ability to properly mimic the tumor microenvironment, test targeted therapies and predict treatment response will improve in parallel. While further research is required prior into the introduction of organoid screening in the clinic, it has unquestionably increased our ability to understand PDAC biology and will greatly aid the search for new treatment opportunities for PDAC patients.
